# Pre-pregnancy characteristics, medical conditions and prenatal support among pregnant women in same-sex relationships: a population-based cohort study

**DOI:** 10.1186/s12978-026-02332-x

**Published:** 2026-04-20

**Authors:** Lisa Kogner, Anna Sara Oberg, Kenny A. Rodriguez-Wallberg, Jonathan M Snowden, Anna Sandström

**Affiliations:** 1 Clinical Epidemiology Division, Department of Medicine, Solna, Karolinska Institutet, Maria Aspmans gata 30A, Stockholm, 17164 Sweden; 2 Division of Obstetrics, Department of Women’s Health, Karolinska University Hospital, Stockholm, Sweden; 3 Department of Medical Epidemiology and Biostatistics, Karolinska Institutet, Stockholm, Sweden; 4 Department of Oncology-Pathology, Laboratory of Translational Fertility Preservation, Karolinska Institutet, Stockholm, Sweden; 5 Division of Gynecology and Reproduction, Department of Reproductive Medicine, Karolinska University Hospital, Stockholm, Sweden; 6 School of Public Health, Oregon Health & Science University-Portland State University, Portland, USA; 7 Department of Obstetrics & Gynecology, Oregon Health & Science University, Portland, USA

**Keywords:** Same-sex relationships, Pregnancy, Pre-pregnancy health, Obstetric risk factors, Maternal health, Reproductive equality

## Abstract

**Background:**

Limited research exists on the obstetric risk profiles of women in same-sex relationships (WSSR) undergoing childbirth.

**Methods:**

Population-based cohort study including deliveries in the Swedish Stockholm-Gotland regions, January 2008-June 2020. Linkage with national health and quality registers provided data on relationships status. Characteristics of WSSR were compared with those of women in different-sex relationships.

**Results:**

Of the 289 979 included deliveries, 1 292 (0.45%) were to WSSR. Compared to women in different-sex relationships, WSSR were more likely to be older, nulliparous, of Swedish origin, and have higher educational attainment. Assisted reproduction was reported for 87.5% of WSSR, versus 7.2% in the comparison group. Smoking during pregnancy was less common for WSSR (1.2% vs. 3.8%). Adjusting for parity and age, WSSR were more likely to be overweight in early pregnancy (adjusted odds ratio [aOR] 1.47; 95% CI 1.30–1.67). Furthermore, they were more likely to have received support for fear of childbirth (12.2% vs. 9.7%; aOR 1.48 95% CI 1.14–1.91). Self-rated pre-pregnancy health and history of major medical conditions were largely similar across both groups, although WSSR were more likely to have been diagnosed with thyroid conditions, rheumatic diseases, gynecological conditions and psychiatric illnesses.

**Conclusions:**

When undergoing childbirth, WSSR more often presented with obstetric risk factors including higher age, nulliparity, and overweight. However, they were also more likely to have higher educational attainment and less likely to report smoking. Pre-pregnancy medical conditions were largely similar between groups. Even so, psychiatric disease and support for fear of childbirth were more common in WSSR, highlighting the need for targeted support during pregnancy.

**Supplementary Information:**

The online version contains supplementary material available at 10.1186/s12978-026-02332-x.

## Abstract (plain language summary)

This study explored the health and background of women in same-sex relationships when they become pregnant. Research on this group has been limited, even though the number of families formed by same-sex couples is increasing.

We used data from almost 290,000 births in the Stockholm-Gotland regions in Sweden between 2008 and 2020. By linking national health and population registers, we identified women in same-sex and different-sex relationships and compared their characteristics before and during pregnancy.

Women in same-sex relationships were generally older, more often first-time mothers, and more likely to have a university education. Most conceived through assisted reproduction. They were less likely to smoke but more likely to be overweight at the start of pregnancy. They also more often received professional support for fear of childbirth.

Overall health before pregnancy was similar between the groups, but thyroid problems, rheumatic diseases, gynecological conditions, and mental health issues were somewhat more common among women in same-sex relationships.

These findings suggest that while many health behaviours are positive, women in same-sex relationships may face specific challenges and would benefit from tailored support during pregnancy and childbirth.

**Supplementary Information:**

The online version contains supplementary material available at 10.1186/s12978-026-02332-x.

## Introduction

Women in same-sex relationships (WSSR) are a growing part of the pregnant population, both in Sweden and globally. Access to marriage and family building services differs around the world, and even though advancements in equality have been made in the last decades, individuals in same-sex relationships still face disparities and discrimination. In Sweden, a partner act was introduced in 1995, and in 2009 same-sex marriage became legal. Due to social reasons, i.e. need for sperm to achieve conception, WSSR largely rely on assisted reproduction to achieve pregnancy. Since 2005 WSSR have the same legal rights to publicly financed assisted reproduction (intrauterine insemination, IUI, and in-vitro fertilization, IVF) as different-sex couples in Sweden.

General health studies of non-pregnant sexual minority women (i.e., lesbians, bisexuals) indicate higher health risks than heterosexual women, including obesity (body mass index [BMI] > 30 kg/m^2^), elevated BMI [1], smoking [2, 3], illicit drug use, and risky drinking habits [4–6]. Numerous studies show that they also have a higher burden of psychiatric illness and suicidal behavior [6–10]. Although these women report poorer health in survey studies [6, 11], data are mixed regarding hypertension, cardiovascular diseases and diabetes [10, 12].

Studies suggests that WSSR may have lower utilization of preventive healthcare and routine gynecological examinations [3, 13, 14]. However, literature on the gynecological health of WSSR is limited and often characterized by methodological limitations, including small sample sizes and recruitment of patients undergoing fertility treatment. The potential increased risk of PCOS among sexual minority women compared to heterosexual women remains debated, with studies reporting mixed findings. Nevertheless, a systematic review published in 2017 found no differences in the prevalence of the fertility-related gynecological conditions PCOS, endometriosis and uterine fibroids [15].

Minority stress is a proposed mechanism underlying sexual orientation health disparities. Stress from stigmatization, marginalization and discrimination may contribute to adverse health outcomes at the population level [16, 17]. The extent and impact of minority stress depends on social and cultural context, such as societal acceptance, legal rights and recognitions of sexual minorities, resulting in differences in sexual orientation-based disparities across populations and nations. Sweden is generally characterized by a high degree of legal recognition and social acceptance of sexual minority individuals [18]. Minority stress may operate differently in this context with potentially less, or other, differences between WSSR and heterosexual women than in studies from other populations and nations.

Research on the reproductive health of WSSR remains scarce, and subject to several methodological challenges. The collection of sexual orientation data has historically been lacking in medical records and public health surveys, hindering identification and accurate assessment of representative populations of sexual minority individuals. To date, study samples have often been small, and lacking in diversity with regard to geographic location, race/ethnicity and socioeconomic background. U.S. studies suggest higher prevalence of obstetric risk factors such as smoking [19, 20], higher age and BMI [21] among pregnant WSSR. In contrast, a Swedish study of children born by WSSR in 1995 to 2010 found less maternal smoking, similar BMI and more favorable socioeconomic status, compared to mothers in different-sex couples [22]. Compared to single women, WSSR had similar BMI and smoking habits but were younger, in a Swedish study of women undergoing assisted reproduction [23].

Maternal stress during pregnancy has been associated with adverse offspring outcomes, including small for gestational age (SGA) birth and preterm birth [24–26]. Minority stress may contribute to these risks among WSSR. Emerging evidence also suggests that WSSR may have higher risk of gestational hypertension and preeclampsia [27, 28], postpartum hemorrhage, and severe maternal morbidity during pregnancy and childbirth [28], as well as increased risk of preterm birth and low birthweight [20, 29]. However, findings are inconsistent, with some studies reporting no differences [21, 23]. Further research, particularly in settings with potentially different levels of minority stress, is needed to clarify these associations. In order to understand whether observed associations could be due to systematic differences in underlying risk factors for adverse pregnancy and delivery outcomes, the pre-pregnancy characteristics and medical conditions among WSSR need to be studied in a first step.

To address this knowledge-gap, we conducted a population-based study in the Swedish Stockholm-Gotland Perinatal Cohort (SGPC), linked to population registers with information on gender of the non-birthing partner. Identifying sexual minority individuals in databases remains a global challenge, and this study provides a unique opportunity to improve the understanding of the health status of pregnant WSSR [30]. The aim was to determine whether pre-pregnancy risk factors associated with adverse obstetric and neonatal outcomes differ between WSSR and women in different-sex couples, using a primarily descriptive approach.

## Methods

### Study design and data sources

This cohort study utilizes the population-based SGPC, a database of pregnancies and deliveries from gestational week 22 + 0 in the Stockholm-Gotland regions in Sweden with registered births between January 2008 to June 2020, based on data retrieved from the medical record system Obstetrix [30]. Information is systematically collected throughout pregnancy, from antenatal visits, to delivery, postnatal care and out-patient care follow-up. Data are automatically and prospectively retrieved from the medical records and contain granular information on both maternal background, reproductive history, and surveillance during pregnancy and delivery.

The cohort was linked to multiple national registers using the Swedish personal identity number [31]. From 2014, additional antenatal care data were obtained from the Swedish Pregnancy Register [32]. The National Quality Registry for Assisted Reproduction (Q-IVF) [33], provided information on assisted reproductive interventions conducted in the Stockholm-Gotland regions since 2007. Statistics Sweden supplied data from the Total Population Register [34] and Multi-Generation Register on cohabitation, marriage and partnership for the delivering women, as well as the biological parents, registered caregivers, and adoptive parents for the infants. Education level was obtained from the Swedish Educational Register. Diagnoses according to the Swedish version of International Classification of diseases 10th edition (ICD-10) were retrieved from inpatient and specialized outpatient care from the National Patient Register, and prescribed medications from the Swedish Prescribed Drug Register, National Board of Health and Welfare. All data were pseudo-anonymized prior to analysis; the personal identification numbers were replaced with serial numbers by the Swedish National Board of Health and Welfare and the identification key was not available to the researchers. Informed consent was not required.

### Study population

**Fig. 1:**
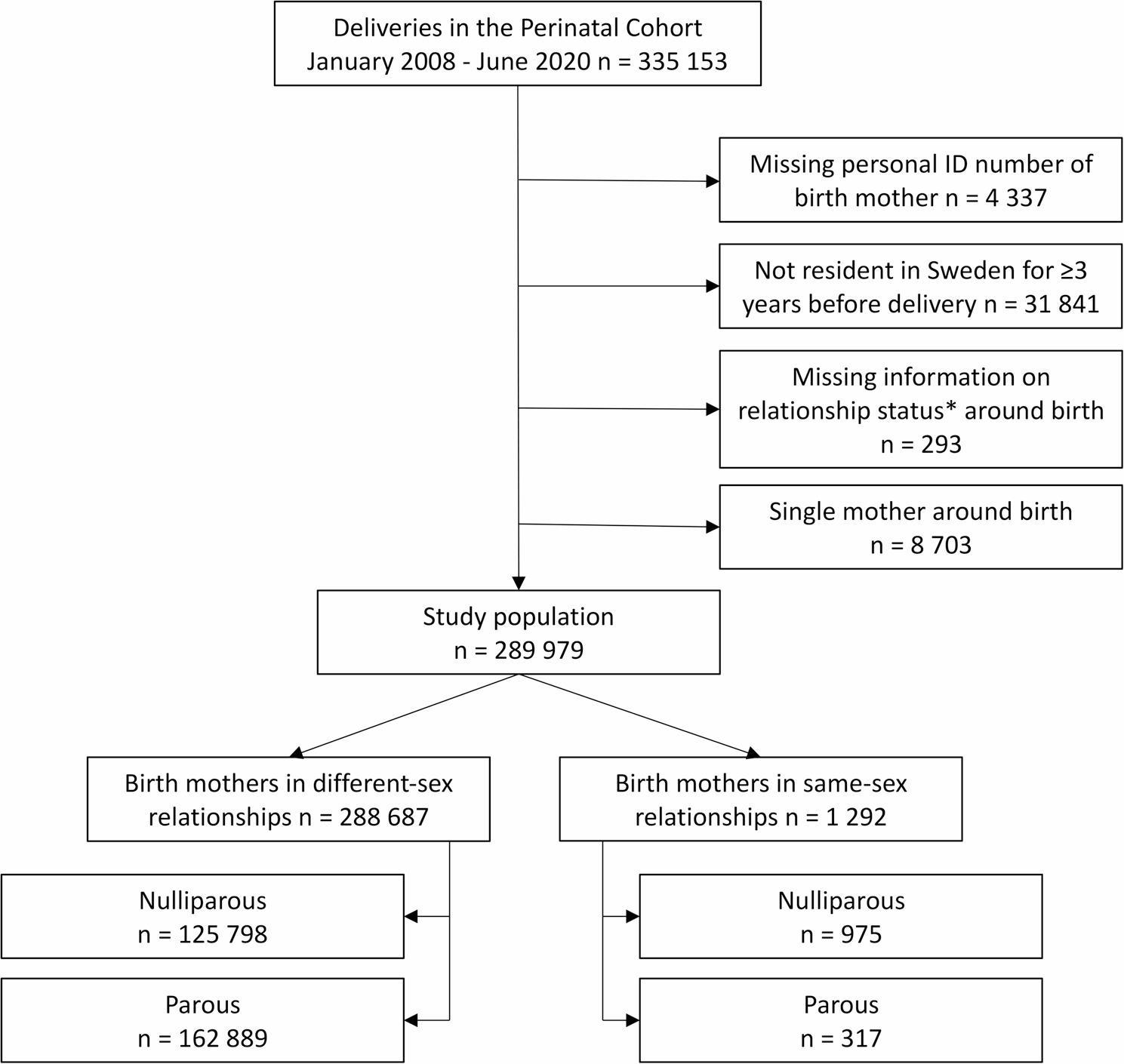
Flowchart of the study population. *According to sources and hierarchy described in Figure 2

This study covers live and stillbirths from gestational week 22 + 0 in the Stockholm-Gotland regions between January 2008 to June 2020, limited to births with an available personal identity number of the birthing mother. Mothers were required to have been residents in Sweden for at least three years prior to delivery to enable assessment of prior inpatient and outpatient healthcare in the Patient Register. Study population selection is presented in Fig. 1.

### Comparison groups: identifying women in same-sex and different-sex relationships

The comparison of interest was relationship status of the mother around the time of delivery, which provided insight into maternal sexual minority status. These data sources did not provide sufficient information to capture the nuances of sexual orientation of each birth mother. Rather, her relationship status around pregnancy and delivery provides a proxy for sexual orientation. The group of WSSR can be inferred to be sexual minority women (i.e., lesbians, bisexuals), although sexual orientation cannot as readily be inferred for women in different sex relationships which may include bisexual women or heterosexuals.

Relationship status was categorized into either different-sex or same-sex relationship. To ensure accuracy, a hierarchal approach was applied to determine relationship status based on the following data sources: (1) *For the birth mother*: cohabitation status and sex of registered spouse or partner from the Total Population Register; sex of registered partner in the Q-IVF Register; and cohabitation status registered during antenatal care in the SGPC; (2) *For the infant*: registered biological parents, caregivers or adoptive parents from the Multi-Generation Register.

**Fig. 2:**
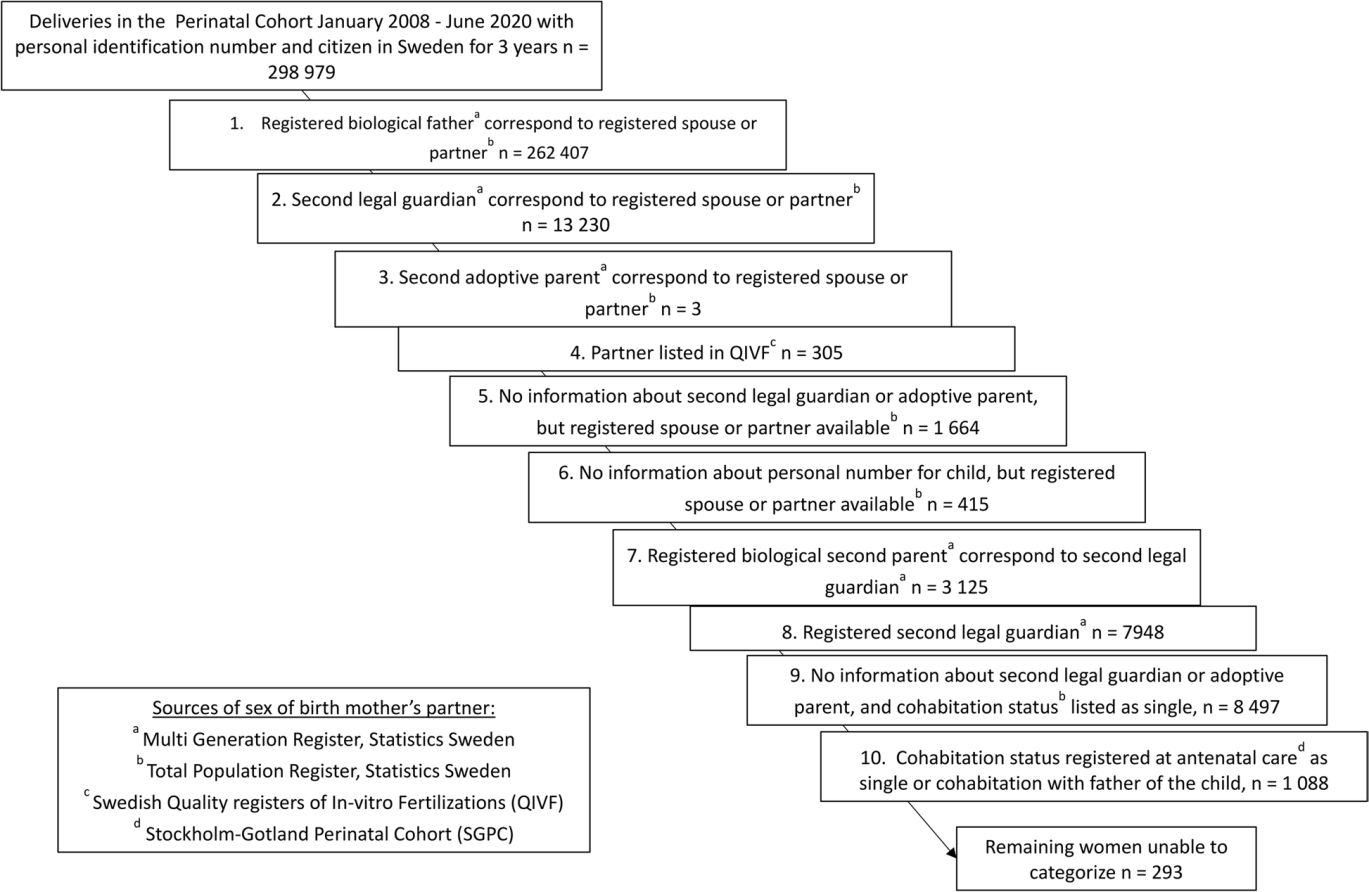
Hierarchy for identification of relationship status of birth mothers in the study population of 289 979 deliveries in the in the Stockholm-Gotland Regions January 2008 - June 2020

Relationship status was determined through a ten-step hierarchical algorithm based on the sources described above, and detailed in Fig. 2. Briefly, the first step compared the pseudonymized numbers of the infant’s listed biological father with the number of the registered partner/spouse of the birth mother, and if they matched, the partner’s sex was male. The second and third steps compared the infant´s second legal guardian or adoptive parent with the registered partner or spouse, and if they matched, the partner’s sex was assigned. The fourth step used partner’s sex data from the Q-IVF. Women categorized as single or with unknown relationship status were excluded, giving only WSSR and women in different-sex relationship. A woman could contribute multiple pregnancies, with relationship status determined separately for each.

### Maternal characteristics, medical conditions and additional variables of interest

From the SGPC we obtained information about birth mothers’ age, parity, BMI at first antenatal visit, smoking, snuff and alcohol use during pregnancy, prior miscarriage or stillbirth, gestational age at first antenatal visit, and number of antenatal visits to midwife. Country of birth was collected from the Total Population Register, since previous research has shown elevated risk of adverse perinatal outcomes among foreign-born mothers in Sweden [35, 36]. Education level at the year of delivery was provided from the Swedish Educational Register.

Women in same- versus different-sex relationships often follow different pathways to pregnancy, which itself may affect pregnancy health [37]. Information on use of IVF and IUI performed in the regions was obtained from the Q-IVF. Additional information was collected from self-reports at the first antenatal visit recorded in the SGPC, as well as ICD-10 codes in the SGPC and Patient Register (described in detail in Supplemental Table S1) to identify treatments performed elsewhere in Sweden or abroad.

Pre-pregnancy medical conditions included chronic hypertension, cardiovascular disease, diabetes mellitus type 1 or 2, rheumatic disease, thyroid disorder, inflammatory bowel disease (IBD), venous thromboembolism, epilepsy, gynecological conditions (polycystic ovary syndrome, endometriosis, myoma), psychiatric disorders, neuropsychiatric disorders, alcohol abuse and drug abuse. Conditions were identified by ICD-10 codes in the Patient Register. For certain conditions, additional information was obtained from the SGPC or from the Swedish Prescribed Drug Register, using ATC (Anatomical Therapeutic Chemical) codes. Definitions and identification algorithms are provided in Supplemental Table S1. Overall healthcare utilization before pregnancy was assessed based on the number of inpatient or specialized outpatient care in the Patient Register three years prior onset of pregnancy. For this, visits to gynecological or obstetric units were excluded, identified through medical division codes, to avoid counting visits due to fertility treatments or previous deliveries.

From the Swedish Pregnancy Register, we retrieved information on antenatal doctor visits for pregnancy-related reasons, pre-pregnancy self-rated health, and participation in parental support during pregnancy (e.g., attending lectures, group discussions or other forms) for the birth mother and/or partner. Data on additional consultations specifically for support related to fear of childbirth (including additional midwife visits, consultation with a doctor or psychiatrist/social worker) was also obtained. These variables were manually entered in the Swedish Pregnancy Register by the midwife in antenatal care, based on the woman’s self-report or information from the medical record. As the register includes births from 2014, and early years had substantial missing information for these variables, analyses of these outcomes were restricted to deliveries from 2016 and onwards.

### Temporal trends

The number and proportion of deliveries to WSSR for each year of the study period were presented graphically, to illustrate temporal change. Additionally, the temporal trend for maternal age, parity, and self-rated health were explored. To better capture shifts in these characteristics, rather than fluctuations, we grouped the study period by 3-year intervals (1 = 2008–2010, 2 = 2011–2013, 3 = 2014–2016, 4 = 2017-June 2020). Due to its limited availability, self-rated health was only evaluated from 2016 to 2020. Potential differences over time were tested using chi-square test for parity and self-rated health, and one-way ANOVA for maternal age.

### Statistical analyses

Distributions of pre-pregnancy characteristics and medical conditions were analyzed separately for women in same and different-sex relationships. Categorical variables were summarized using counts and frequencies, and continuous variables using medians and interquartile ranges (IQR). The primary aim of this study was to descriptively compare the health status of women undergoing pregnancy and delivery by relationship status, without inferring causality. Some variables (e.g., IVF) are better conceptualized as mediators rather than confounders. Nevertheless, to explore associations, we performed logistic regression for binary outcomes and multinomial logistic regression for categorical outcomes to estimate crude odds ratios (OR) with 95% confidence intervals (CI) between the groups. Given that age and parity largely differed between the groups and are closely associated to other obstetric and neonatal risk factors we also report adjusted OR (aOR), controlling for these two variables, while noting that also this aim to describe differences and not attributable risk. All analyses and data handling were conducted using SAS version 9.4 (SAS Institute, Cary, NC).

## Results

**Fig. 3:**
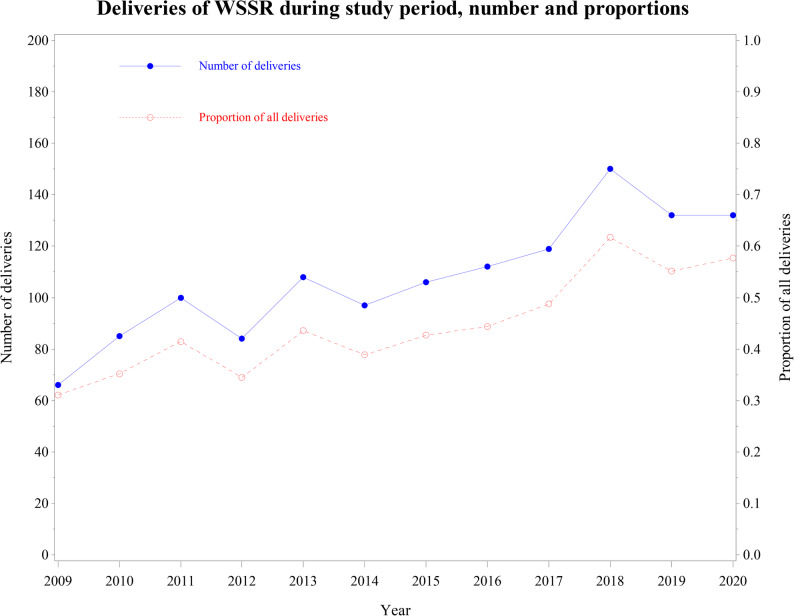
Temporal trends in deliveries by women in same-sex relationships in in the Stockholm-Gotland Regions January 2008 - June 2020, presented by the number and proportions per year, defined from July 1 of the previous year to June 30 of the current year

From the initial cohort of 335 153 deliveries, the final study population included 289 979 deliveries to 193 385 unique birth mothers. Of these, 288 687 deliveries were to women in different-sex relationships (192 310 unique birth mothers), and 1 292 (0.45%) to 1 095 WSSR. Study population selection is shown in Fig. 1. Among WSSR, 75.5% were nulliparous, compared to 43.6% of women in different-sex relationships. The number of deliveries to WSSR increased over the study period, both in absolute terms and as a proportion of all deliveries (Fig. 3).

Distributions of pre-pregnancy characteristics, medical conditions and support during pregnancy are presented in Table 1. The group of WSSR were older, with a median age of 34 (IQR 31–37), compared to 32 (IQR 29–35) years for women in different-sex couples. Nulliparity was more common among WSSR, with only 2.7% expecting their third child or more, compared to 17.7% among women in different-sex relationships. Median BMI was similar between WSSR and women in different-sex relationships (23.7 compared to 23.2). However, WSSR were more likely to be overweight (BMI 25.0-29.9; aOR 1.47 95% CI 1.30–1.67), but not obese (BMI ≥ 30; aOR 1.18 95% CI 0.97–1.44). Furthermore, WSSR were more often of Swedish origin and had a higher educational level in early pregnancy (Table 1).

**Table 1:** Pre-pregnancy characteristics and medical conditions among birth mothers, by relationship status in the study population of 289 979 deliveries in the Stockholm-Gotland Regions January 2008 -June 2020

Characteristic	Birth mothers in different-sex relationships *n* = 288 687		Birth mothers in same-sex relationships *n* = 1 292		Odds ratios			
	*n*	(%)	*n*	(%)	OR^a^	(95% CI)	aOR^a, b^	(95% CI)
**Maternal age (years)**								
≤ 24	20 254	(7.0)	15	(1.2)	0.20	(0.12–0.34)	0.15^c^	(0.09–0.26)
25–34	183 569	(63.6)	675	(52.2)	Ref.		Ref.	
35–39	68 595	(23.8)	495	(38.3)	1.96	(1.75–2.21)	2.85^c^	(2.53–3.21)
≥ 40	16 269	(5.6)	107	(8.3)	1.79	(1.46–2.20)	2.77^c^	(2.25–3.41)
Median (IQR)	32	(29–35)	34	(31–37)				
**Parity**								
Nulliparous	125 798	(43.6)	975	(75.5)	Ref.		Ref.	
Parous 1–2	111 916	(38.8)	282	(21.8)	0.33	(0.29–0.37)	0.25^d^	(0.22–0.29)
Parous ≥ 3	50 973	(17.7)	35	(2.7)	0.09	(0.06–0.12)	0.05^d^	(0.04–0.08)
**BMI (kg/m** ^**2**^ **)**								
< 18.5	7 443	(2.6)	9	(0.7)	0.29	(0.15–0.56)	0.32	(0.17–0.62)
18.5–24.9	181 338	(62.8)	765	(59.2)	Ref.		Ref.	
25.0-29.9	62 182	(21.5)	349	(27.0)	1.33	(1.17–1.51)	1.47	(1.30–1.67)
≥ 30	27 211	(9.4)	113	(8.8)	0.98	(0.81–1.20)	1.18	(0.97–1.44)
Median (IQR)	23.15	(21.14–26.03)	23.74	(21.78–26.69)				
*Missing*	*10 513*	*(3.6)*	*56*	*(4.3)*				
**Country of birth**								
Sweden	216 161	(74.9)	1 151	(89.1)	Ref.		Ref.	
Europe	24 643	(8.5)	76	(5.9)	0.58	(0.46–0.73)	0.61	(0.48–0.77)
Other	47 654	(16.5)	65	(5.0)	0.26	(0.20–0.33)	0.32	(0.25–0.41)
*Missing*	*229*	*(0.1)*	*0*	*(0.0)*				
**Education level (years)**								
≤ 9	21 871	(7.6)	26	(2.0)	0.34	(0.23–0.51)	0.45	(0.30–0.68)
10–11	20 034	(6.9)	40	(3.1)	0.56	(0.40–0.79)	0.61	(0.43–0.85)
12	65 713	(22.8)	233	(18.0)	Ref.		Ref.	
13–14	40 203	(13.9)	236	(18.3)	1.66	(1.38–1.99)	1.33	(1.10–1.59)
15+	137 812	(47.7)	751	(58.1)	1.54	(1.33–1.78)	1.13	(0.97–1.31)
Missing	3 054	(1.1)	6	(0.5)				
**Smoking** ^**e**^	10 883	(3.8)	15	(1.2)	0.30	(0.18–0.50)	0.42	(0.25–0.69)
Missing	837	(0.3)	5	(0.4)				
**Snuff** ^**e**^	3 266	(1.1)	9	(0.7)	0.61	(0.32–1.18)	0.65	(0.34–1.26)
**Alcohol use** ^**e**^	2 172	(0.8)	6	(0.5)	0.67	(0.30–1.50)	0.59	(0.26–1.31)
*Missing*	*61 340*	*(21.3)*	*358*	*(27.7)*				
**Previous miscarriage**								
0	216 980	(75.2)	1 066	(82.5)	Ref.		Ref.	
1–2	65 581	(22.7)	210	(16.3)	0.65	(0.56–0.76)	0.69	(0.59–0.80)
≥ 3	6 126	(2.1)	16	(1.2)	0.54	(0.33–0.88)	0.55	(0.33–0.90)
**Previous stillbirth**								
≥ 1	1 791	(0.6)	11	(0.9)	1.38	(0.76–2.50)	3.02	(1.65–5.53)
**Fertility treatment**								
No	268 080	(92.9)	161	(12.5)	Ref.		Ref.	
IUI, ovulation stimulation or both	3 084	(1.1)	308	(23.8)	166.29166.29	(136.45-201.08)	140.96	(115.86–171.50)
IVF/ICSI	17 523	(6.1)	823	(63.7)	78.20	(66.00-92.65)	61.17	(51.22–73.06)
**Gestational age at first antenatal visit (weeks)**								
4 + 1–9 + 6	171 897	(59.5)	593	(45.9)	Ref.		Ref.	
10 + 0–19 + 6	110 998	(38.5)	680	(52.6)	1.78	(1.59–1.98)	1.85	(1.65–2.06)
20 + 0–41 + 0	5 200	(1.8)	15	(1.2)	0.84	(0.50–1.40)	0.93	(0.56–1.56)
Median (IQR)	9 + 3	(8 + 1–10 + 5)	10 + 0	(9 + 0–11 + 0)				
*Missing*	*592*	*(0.2)*	*4*	*(0.3)*				
**Antenatal visits to midwife (n)**								
≥ 13	16 786	(5.8)	71	(5.5)	0.94	(0.74–1.20)	0.74	(0.58–0.94)
Median (IQR)	9	(7–10)	9	(8–10)				
**Medical diagnoses**								
Chronic hypertension	3 445	(1.2)	11	(0.9)	0.71	(0.39–1.29)	0.66	(0.36–1.19)
Cardiovascular disease	471	(0.2)	< 5	-	-	-	-	-
Diabetes mellitus type 1 or 2	2 698	(0.9)	15	(1.2)	1.25	(0.75–2.08)	1.23	(0.73–2.04)
Rheumatic disease	845	(0.3)	9	(0.7)	2.39	(1.24–4.62)	2.08	(1.07–4.03)
Thyroid disorder	10 948	(3.8)	61	(4.7)	1.26	(0.97–1.63)	1.76	(1.36–2.29)
IBD	2 184	(0.8)	8	(0.6)	0.82	(0.41–1.64)	0.83	(0.41–1.67)
Venous thromboembolism	978	(0.3)	< 5	-	-	-	-	-
Epilepsy	1 266	(0.4)	5	(0.4)	0.88	(0.37–2.13)	0.94	(0.34–2.26)
Gynecological conditions	14 464	(5.0)	113	(8.8)	1.82	(1.50–2.21)	1.53	(1.26–1.86)
Psychiatric disorders	23 133	(8.0)	127	(9.8)	1.25	(1.04–1.50)	1.37	(1.14–1.65)
Neuropsychiatric disorders	3 586	(1.2)	15	(1.2)	0.94	(0.56–1.56)	1.21	(0.72–2.01)
Alcohol abuse	3 656	(1.3)	14	(1.1)	0.85	(0.50–1.45)	1.19	(0.70–2.03)
Drug abuse	1 870	(0.7)	5	(0.4)	0.60	(0.25–1.44)	0.83	(0.34–1.99)
**Healthcare visits 3 years before pregnancy** ^**f**^ **(n)**								
0–2	210 772	(73.0)	812	(62.9)	Ref.			
3–6	50 552	(17.5)	313	(24.2)	1.61	(1.41–1.83)	1.64	(1.44–1.87)
≥ 7	27 363	(9.5)	167	(12.9)	1.58	(1.34–1.87)	1.56	(1.32–1.85)
Median (IQR)	1	(0–3)	2	(0–4)				

Outcomes derived from the Pregnancy register for deliveries January 2016-June 2020 Total *N* = 108 524								
Characteristic	Birth mothers in different-sex relationship Total *n* = 107 940		Birth mothers in same-sex relationship Total *n* = 584		Odds ratios			
	n	(%)	n	(%)	OR^a^	(95% CI)	aOR^a, b^	(95% CI)
**Self-rated health before pregnancy**								
Good	86 824	(80.4)	477	(81.7)	Ref.		Ref.	
Bad	8 864	(8.2)	44	(7.5)	0.90	(0.66–1.23)	0.90	(0.66–1.23)
*Missing*	*12 252*	*(11.4)*	*63*	*(10.8)*				
**Antenatal visits to doctor**								
≥ 3 visits	23 401	(21.7)	104	(17.8)	0.84	(0.67–1.04)	0.87	(0.70–1.09)
*Missing*	*23 976*	*(22.2)*	*158*	*(27.1)*				
**Attended parental support**	33 155	(30.7)	298	(51.03)	3.59	(2.92–4.41)	1.72	(1.30–2.29)
*Missing*	*22 925*	*(21.2)*	*156*	*(26.7)*				
**Partner attended parental support**	30 521	(28.3)	289	(49.5)	3.71	(3.03–4.54)	1.88	(1.43–2.46)
*Missing*	*22 925*	*(21.2)*	*156*	*(26.7)*				
**Received support for fear of childbirth**	10 425	(9.7)	71	(12.2)	1.42	(1.10–1.84)	1.48	(1.14–1.91)
*Missing*	*22 925*	*(21.2)*	*156*	*(26.7)*				

*IQR* interquartile range, *BMI* body mass index, *IBD* Inflammatory bowel disease, *IUI* Intrauterine insemination, *IVF* In vitro fertilization, * ICSI* Intracytoplasmic sperm injection, *OR* odds ratios, *CI* confidence interval, *aOR * adjusted odds ratios

^a^Reference=women in different sex relationship

^b^Adjusted for maternal age and parity (if not stated otherwise)

^c^Adjusted for parity

^d^Adjusted for maternal age

^e^In either early or late pregnancy, or both

^f^Visits from inpatient clinics and specialized outpatient clinics, visits to gynecological and obstetrical units excluded

Smoking during pregnancy was less common among WSSR compared to women in different-sex relationships (1.2% and 3.8%, respectively), whereas use of snuff or alcohol during pregnancy was similar between the groups. WSSR had a lower prevalence of previous miscarriages (82.5% reporting no prior miscarriage, compared to 75.2% of women in different-sex relationships). In contrast, previous stillbirth was more common in WSSR (0.9% compared to 0.6%, aOR 3.02 95% CI 1.65–5.53).

Pregnancies among WSSR were most commonly achieved using donor sperm through IVF (63.7%), or IUI (23.8%) performed either in natural cycles or with ovulation induction. In contrast, among women in different-sex relationships, 6.1% had conceived with IVF and 1.1% with IUI and/or ovulation stimulation.

The timing of the first antenatal visit were later for WSSR, who were more likely to initiate antenatal care after 10 + 0 weeks of gestation. However, no difference was seen for first visits after 20 + 0 weeks gestation. The median number of midwife visits during pregnancy was 9 in both groups. After adjusting for age and parity, WSSR were less likely to have ≥ 13 number of visits to a midwife than women in different sex relationships (aOR 0.74 95% CI 0.58–0.94).

For most pre-pregnancy medical conditions, including hypertension, diabetes, IBD, venous thromboembolism, epilepsy, and alcohol or drug abuse, no differences were observed between the groups. However, WSSR were more likely to have been diagnosed with thyroid disease (4.7% compared to 3.8%; aOR 1.76, 95% CI 1.36–2.29), gynecological conditions (aOR 1.53, 95% CI 1.26–1.86) and psychiatric disease (aOR 1.37, 95% CI 1.14–1.65). Although a history of rheumatic disease was rare in both groups, it was more common among WSSR (aOR 2.08 95% CI 1.07–4.03). Healthcare utilization before pregnancy, measured by number of healthcare appointments, was also higher among WSSR. Specifically, 12.9% of WSSR had seven or more appointments before pregnancy, compared to 9.5% in the comparison group.

Despite this, WSSR rated their pre-pregnancy health as good as women in different-sex relationships (91.6% and 90.7%, respectively). There was no difference between the groups in having three or more antenatal visits with a physician. Attending parental support sessions was more common among WSSR and their partners, even after adjusting for age and parity. A higher proportion of WSSR received support for fear of childbirth (16.6% compared to 12.3% of different-sex women), with an aOR of 1.48 (95% CI 1.14–1.91).

Temporal trends among WSSR showed no shift in maternal age (*p* = 0.7683), but an increase in the proportion of parous women over time (*p* = 0.0046). Furthermore, the proportion who self-rated their health as good increased over the years 2016 to 2020 (*p* = 0.0025) (Supplemental Table 2).

## Discussion

### Main findings

In this population-based cohort study, WSSR differed from women in different-sex relationships in several pre-pregnancy characteristics, while most health conditions were comparable between the groups. The majority of WSSR pregnancies were achieved through assisted reproduction. WSSR were generally older, more often nulliparous, and more likely to be overweight, all well-established obstetric risk factors. Conversely, WSSR were less likely to smoke during pregnancy, had higher educational level, and were more often of Swedish origin, characteristics associated with lower risk of adverse perinatal outcomes. Self-rated health was equally good in both groups. However, WSSR demonstrated higher healthcare utilization before pregnancy. While the prevalence of most prior medical conditions did not differ, WSSR showed increased risks for thyroid disorders, rheumatic diseases and gynecological conditions. Furthermore, WSSR had higher rates of psychiatric disease before pregnancy and were more likely to receive support for fear of childbirth.

### Interpretation

There is a lack of studies examining obstetric risk factors and outcomes among WSSR. A recently published scoping review [38], highlighted that literature on perinatal health outcomes for sexual minority individuals remains limited, with mixed evidence regarding disparities. Most existing studies were performed in the U.S., and their findings vary depending on study design and selection of population. The demographic profile of WSSR in our study, including higher educational level, a lower proportion of foreign-born individuals, and older age at childbearing, are consistent with two previous studies from different American states [21, 28]. These studies also supported our findings of a higher proportion of overweight among WSSR [21, 28]. In contrast, other U.S. studies [19, 20, 29], using survey data and smaller sample sizes found no differences in age, and lower educational levels and socioeconomic measures among sexual minority mothers. However, these studies did not report on pre-pregnancy BMI or obesity [19, 20, 29].

In general health studies of non-pregnant women, sexual minority women have higher rates of smoking [2, 3], and these findings have been confirmed in some studies during the perinatal period [19–21]. In contrast, this study found that WSSR smoked less during pregnancy. This discrepancy may be attributable to differences in cultural context and healthcare systems between Sweden and the U.S.

Some of the findings in our study may be explained by the higher proportion of pregnancies achieved through fertility treatments among WSSR. For instance, the greater proportion of WSSR enrolling in antenatal care after 10 + 0 weeks gestation may be explained by early monitoring at fertility clinics prior to transitioning to regular antenatal care. Additionally, examinations of gynecological conditions and thyroid disease are part of the mandatory fertility investigation for all individuals seeking fertility services, which may partially explain why WSSR women in our study were more likely to have been diagnosed with these conditions prior to pregnancy.

Most medical conditions, such as hypertension, cardiovascular disease and diabetes, did not differ between the groups, which aligns with findings from previous public health studies [10, 12]. The higher rate of rheumatic disease among WSSR was unexpected based on previous knowledge [39], and further investigation is needed to determine whether this finding is due to chance or reflects an underlying mechanism.

Sexual minority women tend to report poorer self-rated health than their heterosexual counterparts in general health studies [6, 11, 40], and in a U.S. study of pre-pregnancy health [19]. However, in our study, the groups evaluated their pre-pregnancy health equally good. We speculate that this may reflect a “healthy parent” effect around pregnancy, or possibly a lower level of stigma and minority stress in the Stockholm-Gotland region in Sweden, compared to some U.S. states. Indeed, previous research has demonstrated that more protective local policies and same-sex marriage legislation are associated with better health outcomes [41, 42], and similar trends have been observed in studies linking state regulations to perinatal outcomes [43]. Furthermore, the temporal trend of increasing proportion of WSSR with good self-rated health over the last four years of the study period may also be related to improving access to family building services and increasing social acceptance.

A higher burden of psychiatric illness among WSSR is consistent with previous literature [7–9]. In this study, WSSR more often received support for fear of childbirth, a relatively unexplored area among sexual minority birth mothers [44]. This finding may partially be explained by a higher prevalence of preexisting psychiatric disease and nulliparity, and possibly different help seeking behavior or access to care. Even so, a Swedish qualitative study described an additional layer of fear of childbirth among sexual minority individuals, attributed to minority stress and previous negative experiences of healthcare [17].

Moreover, WSSR showed higher healthcare utilization before pregnancy, which may reflect greater medical needs, or different healthcare seeking behavior, though they did not have more antenatal visits during pregnancy. The reasons for this discrepancy are unclear, but WSSR and their partners were more likely to attend parental support sessions. These sessions, offered through antenatal care, are designed to prepare expectant parents and strengthen their ability to care for their newborn. Data on partner support were not available in this study. However, prior research has demonstrated positive effects of partner support, including encouraging healthcare-seeking behavior [45], and influencing health behaviors during pregnancy and pregnancy outcomes [46, 47]. The higher participation in parental support sessions among WSSR in our study may be a positive indicator, potentially supporting the couple’s transition to parenthood [48, 49].

### Strengths

The strengths of this study include the use of a large population-based cohort with prospectively collected, granular data from high-quality registers. The comparison of interest, relationship status of the birth mother around the time of birth, was assessed using information from population registers. This approach, predominantly without self-reported data, as used in many other studies, minimizes information bias related to stigma or reluctance in disclosing sexual orientation. The hierarchy used to determine relationship status was rigorously constructed, combining data from multiple sources to ensure reliability and robustness. For example, if information about the birth mother’s registered partner or cohabitation status was available, this was compared to data about the child’s registered parents or caregivers. If no such information was available, other sources were used in order of reliability.

Several aspects of health were investigated, including both broad and specific measures. The characterization covers both demographic data, health risk factors, general health, healthcare needs before and during pregnancy, and specific medical conditions. Several of these measures are unique to register-based studies, and have been lacking in previous research. Current legislation granting sexual minorities access to family-building options, along with the widespread use of population-based registers in Sweden, provides an exceptional context for this study. These findings contribute valuable knowledge to a field with limited prior research.

### Limitations

This study also has several limitations. Sexual orientation comprises three dimensions – identity, attraction and behavior. Since our study lacked self-assessed measures of sexual orientation, we were only able to capture behavior. Still, it is reasonable to assume that a woman who becomes a parent in a same-sex relationship likely aligns with other dimensions of minority sexual orientation and is likely to self-identify as lesbian or bisexual. Notably, the cohort only comprises WSSR who reached pregnancy and delivery after gestational week 22 + 0. Therefore, the results and conclusions are specific to this population and life stage, and may not be generalizable to the broader lesbian/bisexual population in other contexts. Additionally, we did not have information on gender identity or expression, and the study does not address aspects of gender diversity. Race or ethnicity are not routinely recorded in Swedish registers or healthcare systems, and thus, this study does not explore potential intersections with racial or ethnic disparities.

Moreover, capturing all kinds of family constellations through registers is inherently complex. Although substantial efforts to reduce the risk of misclassification of partnership status by using and combining multiple high-quality data sources, some potential for error remains. Women with stillbirth lack register information from sources linked to the child, and therefore had a higher proportion of missing information on relationship status (17,3% compared to 9,7% for women with livebirths). Even so, we chose to include them to not miss out on valuable data. 

In Stockholm, the Swedish capital, WSSR generally have better access to fertility clinics, LGBTQ-certified maternity care, and may benefit from a more inclusive metropolitan environment, compared to other regions of Sweden. Additionally, the characteristics observed in this study may differ in countries with less progressive legislation and fewer rights for sexual minorities. Therefore, the external validity of these findings is most applicable to similar socio-political and healthcare settings.

Although the study is based on a large cohort, statistical power may be limited for the comparison of rare medical conditions due to the relatively small size of the WSSR group. Importantly, our aim was limited to a description of potential differences, and not to attribute risk. We performed regression analyses, crude and with adjustment for the two most relevant factors (age and parity), to enhance comparability between the groups. This approach was intended to provide an overview of the health situation of WSSR during pregnancy, rather than to identify specific causal pathways.

## Conclusion

In this study WSSR were more likely to undergo childbirth with obstetric risk factors such as higher age, nulliparity, and overweight, compared to women in different-sex relationships. However, WSSR also exhibited lower prevalence of smoking during pregnancy and higher educational attainment. Most pre-pregnancy medical conditions as well as self-rated health did not differ between the groups. Furthermore, WSSR showed higher participation in parental support activities. However, particular attention should be given to psychiatric disease and fear of childbirth within prenatal care, as these factors can have medical implications and significantly influence the individual experience of pregnancy and childbirth.

## Supplementary Information

Supplementary Material 1.

## Data Availability

The datasets generated and analyzed in the current study are not publicly available due to privacy and data protection regulations but are available from the corresponding author on reasonable request.

## Abbreviations

WSSR: Women in same-sex relationships
IUI: Intrauterine insemination
IVF: In-vitro fertilization
BMI: Body mass index
SGPC: Stockholm-Gotland Perinatal Cohort
Q-IVF: The National Quality Registry for Assisted Reproduction
ICD-10: International Classification of diseases 10th edition
IBD: Inflammatory bowel disease
ATC: Anatomical Therapeutic Chemical
IQR: Interquartile ranges
OR: Odds ratios
CI: Confidence intervals
AOR: Adjusted odds ratios
LGBTQ: Lesbian, gay, bisexual, transgender, and queer
