# Supplementary material for: Pre-pregnancy characteristics, medical conditions and prenatal support among pregnant women in same-sex relationships: a population-based cohort study
Source: Reprod Health. 2026 Apr 20;23:90. doi: 10.1186/s12978-026-02332-x (PMC13126960; doi:10.1186/s12978-026-02332-x)
Supplement: Supplementary file 1 — Supplementary Material 1. [file 12978_2026_2332_MOESM1_ESM.docx]

**Supplemental Table S1.** Study definitions and algorithms for pre-pregnancy characteristics and medical conditions in birth mothers.

| Diagnose | ICD10-codes | Algorithm for identification; source, time span and number of visits. H=hospitalization O=outpatient visit |
| --- | --- | --- |
| Self-rated health before pregnancy |  | Good (Very good, Good)  Bad (Bad, Very bad, Neither good or bad) |
| Fertility treatment  IVF/ICSI  Insemination, ovulation stimulation or both | Z312, O268A  Z311, Z318B | QIVF: Date of embryo transfer or insemination.  Patient register: ICD10 codes ≥1 H or ≥1 O recorded between conception (=after last menstrual period) and birth.  SGPC: ICD-codes or tick box data from early pregnancy.  Women meeting IVF/ICSI criteria were classified as IVF; otherwise meeting criteria for insemination or ovulation stimulation were classified accordingly. |
| Chronic hypertension | I10-I13, I15, O10 | Patient register: ≥1 H or ≥1 O before birth, or diagnosis in SGPC ≥1 H recorded during pregnancy |
| Cardiovascular disease  Coronary heart disease  Cerebrovascular disease  Chronic heart disease | I20-I25  I61-I66, I672, I69, G45  I50, I05-I09, I34-I37, I42, I43 | Patient register: ≥1 H or ≥2 O before conception |
| Diabetes mellitus type 1 or 2 | O24.0 O24.1 O24.2 O24.3, E10-E14  ATC-codes: A10A, A10B | Patient register: ≥1 H or ≥1 O before birth, or SGPC  National prescribed drug register: Drug dispensed within 12 months before conception, excluded if Metformin and PCOS diagnosis, but not excluded if PCOS and diabetes diagnosis |
| Rheumatoid disease  Rheumatoid arthritis  Juvenile arthritis  Spondylarthritis  Psoriatic arthritis  Idiopatic inflammatory myopathy  Systemic sclerosis  SLE  Sjögren’s disease  Takayasu´s arterit  Antiphospholipid syndrome (APS) | M05, M06.0, M06.2, M06.3, M06.8 M06.9  M08, M09  M45.9, M46.8, M46.9  L405, M070, M071, M072, M073  M33, G72.4  M34.0, M34.1, M34.8, M34.9  M32  M35.0  M31.4  D686 A-C | Patient register: ≥1 H or ≥1 O at an internal medicine or rheumatology unit before conception |
| Thyroid disorder | E00–E07 | Patient register: ≥1 H or ≥1 O before conception |
| IBD  Chrons disease, Ulcerative colitis | K50, K51 | Patient register: ≥1 H or ≥1 O before conception |
| Venous thromboembolism  Venous thrombosis  Pulmonary embolism  Sinus thrombosis  Other venous thrombosis | I801, I802, I803, I81, I82,  I26  I636, I676 | Patient register: ≥1 H or ≥1 O before conception |
| Chronic kidney disease | N17, N18, N19 | Patient register: ≥1 H or ≥1 O before conception |
| Epilepsy | G40 | Patient register: ≥1 H or ≥1 O before conception |
| Gynecological conditions  PCOS  Endometriosis  Myoma | E28.2  N80  D25 | Patient register: ≥1 H or ≥1 O before conception |
| Psychiatric disorders  Depression  Anxiety disorders  Bipolar disorder  Psychosis/Schizofrenia  Eating disorder  Emotionally unstable personality disorder  PTSD | F32-F34, F38, F39  F40, F41, F42  F30, F31  F11.5, F12.5, F13.5, F14.5, F16.5, F18.5, F19.5, F20, F22-F25, F28, F29  F50.0-F50.3, F50.9  F603  F431 | Patient register: ≥1 H or ≥1 O before conception |
| Neuropsychiatric disorders  ADHD, ADD  Autism | F90  ATC-codes: N06BA01, N06BA0, N06BA04, N06BA09, N06BA12, C02AC02[47]  F84.0, F84.1, F84.5 | Patient register: ≥1 H or ≥1 O before conception  National prescribed drug register: medications dispensed before conception |
| Alcohol abuse | F10 | Patient register: ≥1 H or ≥1 O before conception |
| Drug abuse | F11  F12  F13  F14  F15  F16  F18  F19 | Patient register: ≥1 H or ≥1 O before conception |

**Supplemental table 2.** Temporal trend among WSSR for the characteristics maternal age, parity, and good self-rated health.

|  | *Year group 1*  2008-2010 | *Year group 2*  2011-2013 | *Year group 3*  2014-2016 | *Year group 4*  2017-June 2020 |  | p-value |
| --- | --- | --- | --- | --- | --- | --- |
| Maternal age, mean (SD) | 33.9 (4.2) | 33.9 (4.0) | 34.1 (4.0) | 34.1 (4.0) |  | 0.7683^a^ |
| Parous women, n (%) | 33 (15.7) | 71 (25.1) | 95 (29.4) | 118 (24.8) |  | 0.0046*^b^ |
|  | ***2016*** | ***2017*** | ***2018*** | ***2019*** | ***2020*** |  |
| Good self-rated health, n (%) | 75 (69.4) | 117 (81.8) | 101 (81.5) | 131 (87.9) | 53 (88.3) | 0.0025*^b^ |

*p<0.05; ^a^One-way ANOVA; ^b^Chi-square
